# *Chenopodium Botrys* Essential Oil as A Source of Sesquiterpenes to Induce Apoptosis and G1 Cell Cycle Arrest in Cervical Cancer Cells

**DOI:** 10.22037/ijpr.2019.1100671

**Published:** 2020

**Authors:** Hasan Rezaieseresht, Saeideh Sadat Shobeiri, Arezou Kaskani

**Affiliations:** a *Traditional and Complementary Medicine Research Center, Sabzevar University of Medical Sciences, Sabzevar, Iran. *; b *Cellular and Molecular Research center, Sabzevar University of Medical Sciences, Sabzevar, Iran. *; c *Immunology Research Center, Bu-Ali Research Institute, Mashhad University of Medical Sciences, Mashhad, Iran. *; d *Department of Chemistry, Sabzevar Branch, Islamic Azad University, Sabzevar, Iran.*

**Keywords:** Chenopodium botrys, Essential Oil, Apoptosis, HeLa Cell, Sesquiterpenes

## Abstract

Conducting cell apoptosis pathways is a novel strategy in cancer treatment. This study aimed to explain that *C. botrys* essential oil could induce apoptosis and arrest the cell cycle in HeLa cells. Cytotoxic and apoptogenic effects of the essential oil of Jerusalem-oak (*Chenopodium botrys* L.), which was obtained from the aerial parts of the plant, were evaluated in HeLa cells. Cell viability was assessed by MTT and LDH assays, and the mechanism of cell apoptosis was investigated using flow cytometry. Expression of the apoptosis-related genes was assessed using real-time polymerase chain reaction (PCR). GC-MS analysis of the herbal essential oil revealed 37 components. The major components were α-Eudesmol (16.81%), Elemol acetate (13.2%), Elemol (9.0%), and α-Chenopodiol-6-acetate (7.9%). The essential oil inhibited the growth of HeLa cells and increased the expression of *p21* and *p53*. In addition, essential oil treatment increased the sub-G1 DNA content and induced apoptosis due to the increased *Bax*/Bcl-2 ratio and up-regulation of caspase-3 gene expression. According to the results, *C. botrys* essential oil exhibited anticancer effects through intrinsic apoptosis pathways and arresting cell proliferation.

## Introduction

*Dysphania botrys* (L.) (syn. *Chenopodium botrys* Linn.), the Jerusalem-oak, belong of Chenopodiaceae family (1), widely grows throughout Western Asia, Europe, and North America(2) . Traditionally, *Chenopodium botrys (C. botrys*) is used in the treatment of several conditions, such as coughing, pectoral complaints, abdominal pain, and obstruction(3). The essential oil of *C. botrys* is collected from Southern Serbia and Greece, exhibiting significant bactericidal and fungicidal effects (4). The concentration and composition of *C. botrys* in its essential may vary between 0.08-2% in different regions(5). Based on previous studies, essential oil of *C. botrys* is composed of monoterpenes (camphor, δ-3-carene, fenchone, linalool, menthone, nerol, β-pinene, pulegone, terpineol-4, and thujone) and sesquiterpenes (β-elemene, elemol and β-eudesmol) (6). Furthermore, there are evidences that sesquiterpenes as β-eudesmol are anti-cancer natural products (7).

Apoptosis is defined as cellular self-destruction or programmed cell death. Studies have confirmed the association of apoptosis and cancer. Moreover, growing evidence suggests that the process of neoplastic transformation, progression, and metastasis involve changes in normal apoptotic pathways (8). 

The main apoptosis signaling pathways are extrinsic (death receptor) and intrinsic pathways (mitochondria). The intrinsic pathway is initiated by stimulants such as anticancer drugs, growth factor withdrawal, and hypoxia or via the induction of oncogenes, which leads to the permeabilization of the external mitochondrial membrane and activates the mitochondrial pathway. The mitochondrial pathway is initiated with the release of apoptogenic agents (e.g., cytochrome C) from the mitochondrial intermembrane space into the cytosol. Release of cytochrome C into the cytosol leads to the activation of *caspase-3* gene through the formation of the apoptosome complex (9). 

Bcl-2 family proteins that regulate mitochondria-mediated apoptosis include the upstream of *caspase* activation. In addition, the Bcl-2 family consists of anti-apoptotic proteins (e.g., Bcl-2, Bcl-xL, and Mcl-1) and some pro-apoptotic proteins (e.g., *Bax*, Bad, and Bim). Overexpression of Bcl-2 anti-apoptotic proteins blocks external mitochondrial membrane permeabilization and inhibits apoptosis (10, 11). It has been suggested that a mediator that could inhibit and/or increase the expression of anti-apoptotic proteins might induce apoptosis in cancer cells.

Human cervical cancer is the second common cause of cancer-related deaths among women worldwide (12). A significant proportion of the patients with cervical cancer are irresponsive to chemotherapy due to the drug resistance of tumors or tremendous drug side-effects (12, 13).

Cancer is characterized by the loss of control over cell growth (14). The tumor suppressor protein *p21* (WAF1/Cip1) is a universal cyclin-dependent kinase inhibitor (CDKI), which regulates cell progression through the cell cycle (15). The induction of *p21* has been observed to be straightly mediated by *p53*, causing an arrest in the cell cycle at the G1 phase(16, 17) . 

In cancer cells, *p21* expression is generally independent of wild-type *p53* expression. Increased expression of *p21* is associated with cell proliferation arrest and metastatic suppression(18, 19). As a tumor suppressor protein, *p53* controls plays numerous physiological roles in cell cycle arrest, DNA repair, apoptosis, translation, and differentiation (17, 18). 

There are several experiments on antimicrobial and anti-inflammatory activity of *C. botrys* essential oil(2, 5). Nevertheless, there are no investigations on anti-cancer effect of this essential oil. According to previous reports on anti-cancer effects of eudesmol compound (7) and presence of this sesquiterpene in *C. botrys* essential oil (6), the present study aimed to elucidate that *C. botrys* essential oil could induce apoptosis and arrest the cell cycle in HeLa cells.

## Experimental

*Materials*

*Collection of Plants*

Aerial parts of *C. botrys *were collected at the altitude of 1,200 meters in the north of Sabzevar, located in Razavi Khorasan province in the northeast of Iran in June 2017. The samples were identified by Souzani at the Herbarium of School of Pharmacy, Mashhad University of Medical Sciences in Mashhad, Iran where the voucher specimen of the plant was deposited. After the hydro-distillation of *C.botrys* aerial parts (100 grams) for two hours, the herbal essential oil was prepared using a Clevenger apparatus. Afterwards, the essential oil was passed over anhydrous sodium sulfate in order to obtain a dried, slightly yellow oil. The essential oil was subjected to cytotoxic assay, and the herbal essential oil was subjected to GC-MS analysis.

*Cell culture*

Human cervial carcinoma cell line (HeLa cells) was purchased from Pasteur Institute of Iran. Dulbecco’s Modified Eagle’s Medium (DMEM GIBCO, England) supplemented with 10% fetal bovine serum (FBS, GIBCO, England) and 1% penicillin/streptomycin (100 U/mL of penicillin G, 100 μg/mL of streptomycin, Biosera, England) was used for the HeLa cells. 

## Methods

*Analysis of C. botrys Essential Oil*

The essential oil *of C. botrys* was analyzed using GC/MS apparatus (GC7890N Agilent MS-5975) to characterize the chemical constituents. The system was equipped with an HP-5MS capillary column (30 m×0.25 mm with film thickness of 0.25 μm). The condition of temperature programming was based on the protocol of helium carrier gas, adjusted to a linear velocity of 1 mL/min, and the column had the split ratio of 50:1. The injector temperature was regulated at 260 °C. 

In the next stage, the temperature of the oven column was maintained at 60 °C for four minutes, programmed to 100 °C at 3 °C/min for two minutes, and to 250 °C at 6 °C/min for seven minutes with the electron energy of 70 eV. To observe the positive ions, the analyzer was scanned from the mass of 50 to 550. Additionally, a mixture of homologous series of n-alkanes C9-C23 was used on the HP-5MS column, and the Kovats indices of the chemical constituents were determined. Constituents of the essential oil were characterized by the comparison of their mass spectra with those of the NIST-5 mass spectra library.

*Cytotoxicity Assay*

In the present study, cytotoxic effects of *C. botrys* essential oil on HeLa cell proliferation was evaluated using the 3-(4, 5-dimethylthi-azol-2-yl)-2, 5-diphenyltetrazolium bromide (MTT) assay. The cells were evenly distributed (5×10^3^ cells/well) on 96-well plates (Iwaki, Tokyo, Japan) and incubated in a humidified incubator at the temperature of 37 ℃ with 5% CO2 overnight. 

In the next stage, after optimizing the concentration range for* C. botrys* essential oil from 0-1mg/mL, the cells were treated by *C. botrys* essential oil at the concentrations of zero, 5, 10, 30, 50, 75, 100, 130, 170, and 200 µg/mL (essential oil solved in DMSO and added cell culture medium) and incubated for 24 h. Following that, the medium in each well was replaced with 20 µL MTT (5 mg/mL in phosphate-buffered saline (PBS)) and incubated at the temperature of 37 ˚C for four hours. The purple-blue formazan crystals were dissolved in 100 µL of dimethyl sulfoxide, and the absorbance was measured at the wavelengths of 510 and 630 nm (control wavelengths) on a 96-well plate reader (Thermo Lab Systems, Franklin, MA, USA). Afterwards, the concentrations of the essential oil that achieved 50% mortality (IC50) were calculated. Data analysis was performed using GraphPad Prism (Advanced Graphics Software, Inc., Rancho, Santa Fe, CA, USA).

*LDH Assay*

*Lactate dehydrogenase (*LDH) levels were measured as the marker of membrane disintegration and permeability increase in the supernatant of the treated and control cells. Moreover, the supernatants containing LDH released from the treated and untreated HeLa cells in 96-well plates were assessed based on the reduction of NAD+ at the wavelength of 330 nm by the Deutsche Gesellschaft *Für *Klinische Chemie method using an auto-analyzer (Biotechnica, BT 1500, Rome, Italy) and commercial kit (Pars Azmoon, Tehran, Iran).

*Apoptosis Assay*

Apoptosis of the treated and untreated HeLa cells was measured by flow cytometry using Annexin V-FITC/PI detection kit (BD, Biosciences Pharmingen) in accordance with the instructions of the manufacturer. HeLa cells (2.5×105 cells/well) were treated with various concentrations of the essential oil of *C. botrys* (50 and 80 µg/mL as IC50 and 100 µg/mL) in six-well plates for 24 h. Afterwards, the cells were detached using trypsin and collected and washed twice with cold PBS. 

The cells were resuspended in Annexin V-binding buffer and incubated with 5 µL of Annexin V-FITC and 5 µl of propidium iodide (PI) for 15 min at room temperature in the dark. Apoptotic and necrotic cell populations were determined on FACSCalibur flow cytometer (BD Biosciences, USA), and the data were analyzed using the CellQuest Pro software package (BD Biosciences, USA). PI-/Annexin V- cells were considered as viable cells, early apoptosis was defined in PI-/Annexin V+ cells, and late apoptosis or necrosis was considered in PI+/Annexin V+ cells.

*Cell Cycle Analysis*

The HeLa cells with exponential growth were seeded in six-well plates (2.5×105 cells/well) and treated by the essential oil of *C. botrys* at the concentrations 50, 80, and 100 for 24 h. Subsequently, the cells were collected and incubated with 20 μg/mL of RNase A and 10 μg/mL of PI in the dark for 30 min. After staining, cell cycle phase distribution and hypodiploid DNA were detected by FACSCalibur flow cytometer (BD Biosciences, USA) at the wavelength of 488 nm and analyzed using the FlowJo 3.1 software (FlowJo, Oregon, USA).

*Real-time PCR*

For gene expression analysis, quantitative real-time polymerase chain reaction (PCR) was performed. HeLa cells (2.5×105 cells/well) were cultured in six-well plates and treated with the essential oil of *C. botrys* at the concentrations of 50, 80, and 100 μg/mL. Total RNA extraction was performed using AccuZol™ Total RNA Extraction Solution (Bioneer, Korea) obtained from the treated and untreated cells. In addition, cDNA synthesis was conducted using 2 μg RNA (Takara Bio, Shiga, Japan). 

SYBR Green PCR Master Mix was applied to determine the expression of *Bax*, *Bcl2*, *caspase-3*, *p53*, *p21*, and glyceraldehyde-3-phosphate dehydrogenase (*GAPDH*) Homo sapiens gene. Moreover, quantitative PCR reactions were performed in a 20-µL volume containing 0.5 µL of each of the forward and reverse primers, 2 µL of cDNA, 10 µL of SYBR Green PCR Master Mix, and 7 µL of Diethyl pyrocarbonate water in accordance with the instructions of the manufacturer. 

After the initial 10 min at the temperature of 94 °C as the activation stage, 35 cycles, including 15 seconds of denaturation at 95 °C, 20 seconds of annealing at 60 °C for *p21* and *p53* and 57 °C for *Bax*, *Bcl2*, and *caspase-3*, and 20 seconds of extension at 72 °C, were carried out using BioRad CFX96 Well Real-Time PCR detection system (Bio-Rad Laboratories, Hercules, CA, USA). Primer sequences and fragments sizes are presented in Table 1.

*Statistical analysis*

Statistical analysis were done by using the SPSS 16.0 software package (SPSS GmbH Software, Germany). The data were presented as mean ± SEM. Comparisons were made using the one-way ANOVA (followed by the post-hoc LSD test) and Brown-Forsythe analysis (followed by the post-hoc Dunnett T3 test). P-values less than 0.05 were considered as level of significance. Graph Pad Prism5 software package was used (Inc.; San Diego CA, USA, 2003) for drawing the statistical graphs.

## Result and Discussion

*Chemical Composition of C. botrys Essential Oil*

The essential oil of *C. botrys* was isolated from the dried aerial parts of the plant through a hydro-distillation process with a yield of 0.97% (w/w) based on the dry weight of *C. botrys*. In total, 37 constituents were detected in the herbal essential oil by GC-MS analysis, as well as their composition and retention index. Accordingly, the essential oil of *C. botrys* was composed of monoterpenes (0.92%), oxygenated monoterpenes (2.68%), sesquiterpenes (13.71%), oxygenated sesquiterpenes (64.17%), and other components (4.06%). The major constituents of *C. botrys* essential oil were α-Eudesmol (16.81%), Elemol acetate (13.2%), Elemol (9.0%), and α-Chenopodiol-6-acetate (7.9%). 

*Cytotoxicity Induction by C. botrys Essential Oil*

**Figure 1:**
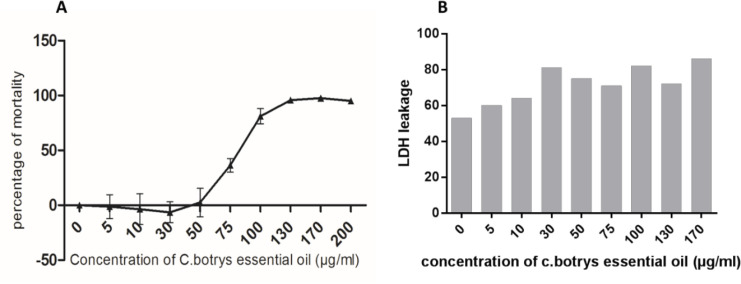
A) Cytotoxic Effects of *C. botrys* on HeLa Cells Measured by MTT Assay; *B)* LDH Leakage Measured in Supernatant of Treated and Control HeLa Cells

In the present study, a broad spectrum of the cytotoxic effects *C. botrys* essential oil on HeLa human cancer cell line was investigated using the MTT assay. Cell viability upon the treatment of cells with increasing concentrations of the herbal essential oil (5-200 µg/mL) was assessed for 24 h. According to the results, the essential oil of *C. botrys* exhibited concentration-dependent cytotoxic effects on the cell line at 24 h. Furthermore, *C. botrys* essential oil exerted significant cytotoxic effects on the HeLa cells with the IC50 of 79.62 µg/mL after treatment for 24 h (Figure 1). 

*Apoptosis Induction by C. botrys Essential Oil in Cervical Cancer Cells*

To determine whether the loss of the cell viability of cervical cancer cells induced by *C. botrys* essential oil was associated with the induction of apoptosis, the HeLa cells were treated by *C. botrys* essential oil, and the number of the apoptotic cells was determined using the Annexin V-FITC and PI detection kit as previously described. 

**Figure 2:**
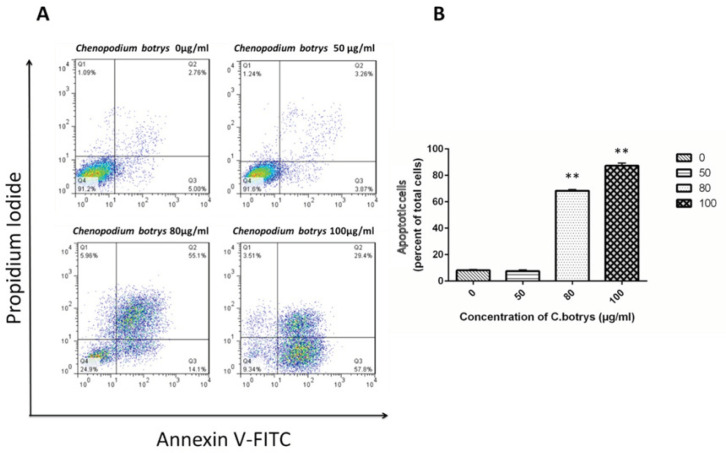
HeLa Cells Treated with *C. botrys* Essential Oil (50, 80, and 100 µg/mL) for 24 H Analyzed by Flow Cytometry for Apoptosis; *A)* Lower right quadrant shows Annexin-V positive cells (early apoptotic), upper left quadrant shows PI positive cells (necrotic cells), upper right quadrant shows Annexin-V and PI positive cells (late apoptotic cells), and lower left quadrant shows PI negative and Annexin-V negative; *B)* Percentage of total apoptosis for each condition is characterized in a histogram. The number of apoptotic cells were significantly increased in 80 µg/mL (*p ˂ 0.001*) and 100 µg/mL (*p ˂ 0.001*) comparing to control group

Apoptotic cells were characterized as early (Annexin V+) and late (Annexin V+/PI+) apoptotic cells. Treatment of the HeLa cells with the essential oil of *C. botrys* led to a significant dose-dependent increase (*p *˂ 0.001) in the number of apoptotic cells from 0 µg/mL to 80 µg/mL and 100 µg/mL, as follows: 0 µg/mL (mean ± SEM: 8.14±0.65), 50 µg/mL (7.53 ± 0.93), 80 µg/mL (68.4 ± 0.91), and 100 µg/mL (87.3 ± 1.95) (Figure 2).

*Induction of Cell Cycle Arrest in G1 Phase in Cervical Cancer Cells by C. botrys Essential Oil*

**Figure 3:**
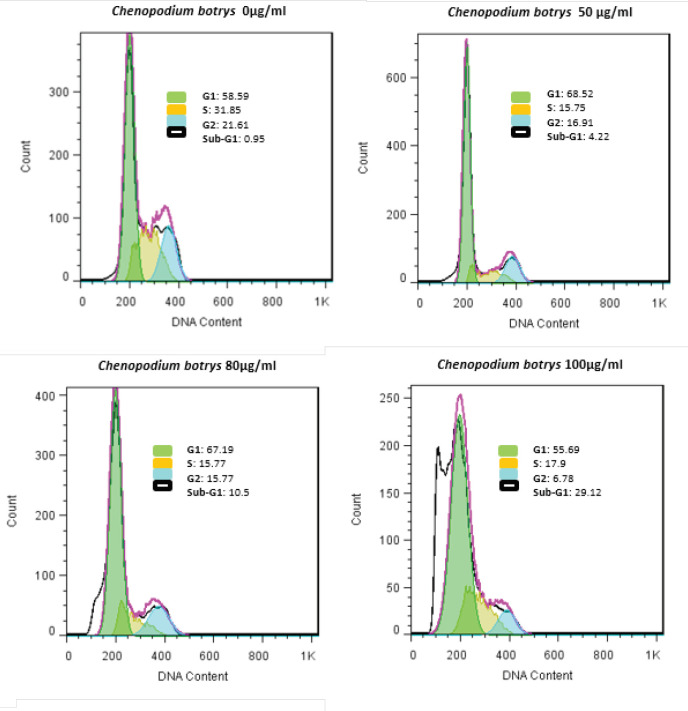
HeLa Cells Treated with *C. botrys* Essential Oil (50, 80, and 100 µg/mL) for 24 H Analyzed by Flow Cytometry for Cell Cycle Assay

Percentage of the cervical cancer cells in various phases of the cell cycle was investigated using the ﬂow cytometry of PI-labeled HeLa cells after 24 h of treatment with *C. botrys *essential oil. Treatment of the HeLa cells by *C. botrys *essential oil caused an increase in the G0/G1 phases and a reduction in the G2 and S phases, which indicated cell cycle arrest in the G1 phase (Figure 3).

*Effects of C. botrys on the Expression of Bcl-2 Family Members and Caspase-3*

**Figure 4:**
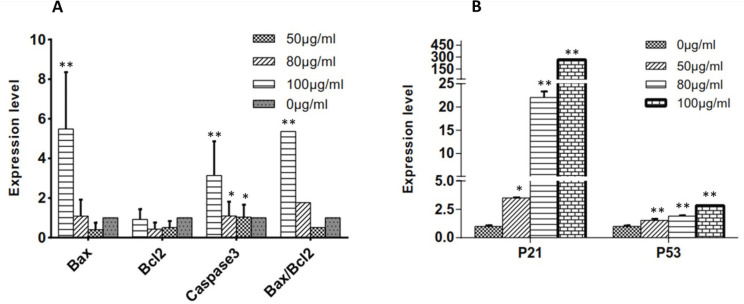
(A) Expression Levels of *Bcl2* Family and *Caspase-3* and *Bax*/*Bcl2* Ratio at Various Concentrations of *C. botrys *Essential Oil (50, 80, and 100 µg/mL); (B) Expression Levels of *p21* and *p53* at Various Concentrations of *C. botrys *Essential Oil (50, 80, and 100 µg/mL). Comparison were done between groups. (**) was considered as *p*value ˂0.001 and (*) was considered for *p*value ˂0.05

To evaluate the status of the intrinsic signaling of apoptosis in mitochondria, mRNA expression levels of the pro-apoptotic (*Bax*) and anti-apoptotic genes (*Bcl2*) were measured using real-time PCR. Moreover, we examined *caspase* involved in *C. botrys*-induced apoptosis by evaluating the mRNA expression level of *caspase-3*. The essential oil of *C. botrys* significantly increased the expression of *Bax* (*p *˂ 0.001, between 0 µg/mL to100 µg/mL) and *caspase-3* (*p *˂ 0.001). Furthermore, the ratio of *Bax*/*Bcl2* had a five-fold increase from the concentration of 50 to 100 µg/mL (*p *˂ 0.001). *Bcl2* gene expression had no significant difference between the groups (Figure 4A).

*Effects of C. botrys Essential Oil on the Expression of p21 and p53*

To assess the status of the HeLa cells in cell growth arrest, we evaluated the expression levels of *p21 *and *p53* genes. According to the findings, the essential oil of *C. botrys* significantly increased the expression of *p21* (*p *˂ 0.001) and *p53* (*p *˂ 0.001) in a concentration-dependent manner, especially in the case of *p21 *(Figure 4B).

Phytochemicals may affect the signaling pathways within the cells, including those involved in the regulation of cell proliferation and activation of apoptosis(20). Apoptosis, also known as programmed cell death, is a pathway through which cells undergo death to control cell proliferation or react to DNA damage (21). Activation of these pathways is a major mechanism, through which cytotoxic drugs eradicate tumor cells (22). In the current research, we evaluated the apoptosis induced by *C. botrys* essential oil in HeLa cells and determined the involved molecular mechanisms that occur via the intrinsic pathway. 

The results of GC/MS analysis showed 37 compounds representing 85.54% of the total contents of the essential oil (Table 2). Moreover, the results indicated that *C. botrys* is an abundant source of monoterpenes, especially sesquiterpenes (78.88%). Several studies have demonstrated that the major compounds of *C. botrys* essential oil are α- and β-eudesmol(23, 24), α- and β-chenopodiol (25), camphor, and elemol (26). However, the main components were noted as α-eudesmol (16.81%), Elemol acetate (13.2%), Elemol (9.0%), and α-Chenopodiol-6-acetate in the current research. In the present study, we investigated the cytotoxic effects of *C. botrys* essential oil on cervical cancer cells, and the herbal essential oil was considered to be a potential cytotoxic agent in the treatment of HeLa cells by killing the cancer cells in a dose-dependent manner. In particular, treatment of the HeLa cells with 100 µg/mL of *C. botrys* essential oil for 24 h could reduce the viability of cervical cancer cells by 81.14 ± 6.87% (IC50:79.64 µg/mL), which indicated the potential to eliminate cancer invasiveness. 

The anti-tumor effects of the herbal essential oil could be attributed to cytostatic and/or cytotoxic function. Cytostatic substances are able to prevent cell growth and/or induce cell cycle arrest at various cell cycle checkpoints. On the other hand, cytotoxic substances induce cell death through apoptosis or necrosis (29). Therefore, we demonstrated a mild elevation in the leakage of LDH by increasing the concentration of the essential oil. Moreover, LDH leakage had a significant adjustment at the IC50 dosage of *C. botrys* essential oil. 

In the current research, apoptosis was followed by cell cycle arrest in the G1 phase, which increased the cells in the sub-G1 (G0) phase and decreased cells in the G2 and S phases (Figures 2 & 3). This finding is supported by the overexpression of *p21* and *p53 *genes. *P21* is known to act as an essential inhibitor of CDK2, which is activated in response to various cellular and environmental signals to improve tumor suppressor events (30). 

In the present study, the wild-type tumor suppressor *p53* and *p21* of the HeLa cells was up-regulated (Figure 4B). Therefore, it could be concluded that the up-regulation of *p53* by *C. botrys* essential oil triggers the accumulation of *p21* protein, leading to G0/G1 phase cell cycle arrest and apoptosis in HeLa cells (31-33). It is also notable that *p53*/*p21* is involved in the control of cell cycle, apoptosis, and maintenance of genomic stability (34). Once DNA damage occurs, wild-type *p53* is expressed to induce cell cycle arrest at various checkpoints in order to repair the damaged DNA. As a result, unrepaired DNA cells are targeted by apoptosis (32). Furthermore, this is accompanied by an increase in the expression of the *Bax* and *Bax*/*Bcl2* ratio. The cytosolic section of *p53* activates the apoptotic-effector protein *Bax* to initiate apoptosis (35). The damaged cells could be repaired to maintain the integrity of the cell or be cleared by apoptosis to remove a cancerous cell through G1 phase arrest. Apoptosis is an aspect of mammalian cell behavior, which is of utmost importance in the growth and development in tumor oncogenesis. 

*Bcl2* and *Bax* are the critical regulators of cell apoptosis with remarkable anti-apoptotic and pro-apoptotic effects, respectively (36). According to the literature, susceptibility to apoptosis is likely to be determined based on the *Bax*/*Bcl2* ratio as the key regulators of apoptosis located mainly at the outer membrane of the mitochondria and endoplasmic reticulum. In the present study, their regulation of apoptosis was mainly achieved through the mitochondria. Overexpression of *Bcl2* leads to resistance to apoptosis, whereas overexpression of *Bax* results in increased apoptosis (36, 37). 

Our findings indicated the adjustment of the expression of *Bcl2* and *Bax* compared to the control group. *Bcl2* expression decreased significantly, while *Bax *expression had a statistically significant increase after treatment with *C. botrys* essential oil. Moreover, the *Bax*/*Bcl2* ratio and *caspase-3* elevated in the HeLa cells after treatment with* C. botrys* essential oil, which is consistent with the results regarding cell apoptosis. 

According to the results of the present study, *C. botrys* essential oil induced intrinsic apoptosis through adjusting the expression of *Bcl2* and *Bax* to enhance their ratio, followed by the triggering of the *caspase* cascade. Therefore, it could be concluded that *C. botrys *essential oil could potentially eliminate cancer cells by conducting apoptosis pathways.

**Table 1:** Primer Sequences

**Gene**		**Sequence**	**bp**
***Bax***	Forward	CGCCCTTTTCTACTTTGCCA	166
	Reverse	GTGAGGAGGCTTGAGGAGTC	
***Bcl2***	Forward	GCCTTCTTTGAGTTCGGTGG	192
	Reverse	GAAATCAAACAGAGGCCGCA	
***Caspase-3***	Forward	ACTGGACTGTGGCATTCAGA	162
	Reverse	GCACAAAGCGACTGGATGAA	
***p53***	Forward	CCCCTCCTGGCCCCTGTCATCTTC	265
	Reverse	GCAGCGCCTCACAACCTCCGTCAT	
***p21***	Forward	CGCATGGGTTCTGACGGACATCC	257
	Reverse	TGCCGAAGTCAGTTCCTTGTGG	
***GAPDH***	Forward	GGAAGGTGAAGGTCGGAGTCA	101
	Reverse	GTCATTGATGGCAACAATATCCACT	

**Table 2:** Chemical Composition of C. botrys Essential Oil

**Class**	**RIL3**	**RIL2**	**RIL1**	**Kovats Retention Index** (RI^a^**)**	** %**	**Rt (min)**	**Compounds**	**No.**
MH	991	990	981	981.8085	0.60	4.986	β-Myrcene	1
MH	1031	1030	1014	1017.147	0.32	6.143	Limonene	2
MO			1096	1073.13	0.97	8.239	α-Thujone	3
MO		1145	1124	1126.591	0.22	10.377	p-Menth-2-en-1-ol	4
MO	1177	1180	1169	1162.908	0.35	11.923	4-Terpineol	5
MO		1193	1187	1177.402	0.52	12.54	α-Terpineol	6
MO	1285		1285	1270.805	0.36	16.582	Bornyl acetate	7
MO	1354	1354	1354	1336.388	0.26	19.607	citronellyl acetate	8
SH	1391	1395	1382	1378.25	2.64	21.626	β-elemene	9
SH		1425	1420	1398.155	1.42	22.586	Trans-caryophyllene	10
SH		1460	1451	1433.083	0.28	23.955	α-Humulene	11
SO			1454	1442.879	0.23	24.334	Trans-geranylacetone	12
SH			1470	1465.96	0.52	25.227	β-Eudesmene	13
SH			1485	1484.053	1.13	25.927	Aromadendrene	14
SH			1505	1494.908	1.40	26.347	γ-Cadinene	15
SH	1524		1516	1505.52	0.45	26.726	δ-Cadinene	16
SO			1539	1517.956	0.68	27.136	α-Copaen-11-ol	17
SO	1540		1547	1537.307	9.02	27.774	Elemol	18
SH	1556		1562	1551.319	0.85	28.236	Germacrene B	19
SO		1583	1578	1561.541	0.95	28.573	Caryophyllene Oxide	20
SO		1644		1624.721	0.27	30.571	γ-Eudesmol	21
SO		1653		1641.754	16.81	31.074	α-Eudesmol	22
SO			1675	1667.931	13.17	31.847	Elemol acetate	23
SO		1678		1673.383	1.40	32.008	botrydiol	24
SO		1691	1682	1678.835	1.13	32.169	juniper camphor	25
SO			1729	1696.749	0.42	32.698	α-Costol	26
SO		1724	1736	1701.249	0.55	32.828	guaiol acetate	27
SO	1778		1782	1770.242	5.05	34.706	γ-eudesmol acetate	28
SO	1789		1785	1779.023	2.44	34.945	α-eudesmol acetate	29
SO			1791	1788.758	4.53	35.21	β-Eudesmol acetate	30
SO		1830		1866.967	4.30	37.223	β-Chenopodiol	31
SO		1876		1875.52	0.38	37.441	α-Chenopodiol	32
SO		1904		1883.837	0.55	37.653	β-Chenopodiol-6-acetate	33
NH			1928	1917.371	0.66	38.484	Methyl Palmitate	34
SO		1957		1930.473	2.15	38.8	Acetoxyeudesman-4-α-ol-11	35
SO		1977		1940.796	7.88	39.049	α-Chenopodiol-6-acetate	36
NH			2128	2113.458	0.68	43.065	Methyl Stearate	37
					85.54		Total Identified Chemicals	
					0.92		Monoterpenes (MH) (%)	
					2.68		Oxygenated Monoterpenes (MO) (%)	
					13.71		Sesquiterpenes (SH) (%)	
					64.17		Oxygenated Sesquiterpenes (SO) (%)	
					1.34		Non terpene hydrocarbon (NH)	

RI^a^: calculated retention index relative to C9-C23 n-alkanes on HP-5MS column, RIL1 (NIST Chemistry Webbook, edited by P. J. Linstrom and W. G. Mallard, available at http://webbook.nist.gov), RIL2(27) literature retention indices on HP-5MS Column, RIL3 (28) literature retention indices on DB-5 column.

## Conclusion

According to the results, *C. botrys* essential oil induced cell cycle arrest in the HeLa cells via the *p53*/*p21* pathway. In addition, the essential oil induced intrinsic apoptosis by increasing the *Bax*/*Bcl2* ratio. Sesquiterpenes were the major compounds in the essential oil of *C. botry*. It is suggested that *C. botrys* essential oil may be a potential candidate for management of cancer; however further researchs are needed to confirm this hypothesis
